# ‘One feels somewhere that one is insignificant in that system’ – older multimorbid patients’ between lifeworld and system in healthcare

**DOI:** 10.1186/s12877-021-02348-x

**Published:** 2021-06-29

**Authors:** Lilian Keene Boye, Christian Backer Mogensen, Pernille Tanggaard Andersen, Frans Boch Waldorff, Thorbjørn Hougaard Mikkelsen

**Affiliations:** 1 Emergency Department, Hospital Sønderjylland, Kresten Philipsens vej 15, indgang F, 6200 Aabenraa, Denmark; 2 Research Unit of Emergency Medicine, Department of Regional Health Research, University of Southern Denmark, Odense, Denmark; 3 Unit of Health Promotion, Department of Public Health, University of Southern Denmark, Esbjerg, Denmark; 4 Research Unit of General Practice, Department of Public Health, University of Southern Denmark, Odense, Denmark; 5 Research Unit for General Practice and Section of General Practice, Department of Public Health, University of Copenhagen, Copenhagen, Denmark

**Keywords:** Life world, System, Habermas, Continuity of care, Older people, Acute hospitalization

## Abstract

**Background:**

When older multimorbid people are acutely hospitalized, continuity of care is a fundamental goal in the healthcare process. However, some acute hospitalized older multimorbid patients do not experience continuity of care. This phenomenon is explored using the theoretical framework of Jürgen Habermas “Theory of communicative action”.

**Methods:**

Acutely hospitalized patients over the age of 65 with two or more chronic conditions and who received home care services were invited to participate in two interviews: one at the emergency department and the other 4–12 weeks after discharge. These interviews formed the basis for an evaluation of patient experience of continuity of care, and the content of the interviews was analyzed using a structured matrix.

**Results:**

Fifteen patients participated with seven patients evaluated to have continuity of care in their healthcare process. Eight patients were evaluated as not having experienced continuity of care in their healthcare process. The categories from the matrix highlighted a healthcare system that interfered with a patient’s lifeworld with a lack of communication, different expectations, frustration regarding care, strained relations to health care providers and feelings of being objectified.

**Conclusions:**

We conclude that mutual understanding based on communicative action is essential when it comes to patients’ experiences of continuity of care. Our results justify improving the mutual understanding between patients and professionals in transition between healthcare sectors. Future research should target whether an enhanced focus on communicative action and mutual understanding in particular between non-healthcare professionals and patients will improve the patients’ perception of continuity of care.

**Supplementary Information:**

The online version contains supplementary material available at 10.1186/s12877-021-02348-x.

## Background

Healthcare systems are multifaceted and complicated [1, 2]. Experiencing and navigating a healthcare system can be challenging, especially for patients with complex health problems [3]. The likelihood of being acutely admitted to hospital increases with age [4, 5]. Continuity of care is a fundamental objective in the healthcare process, including when a patient is acutely admitted. Continuity of care is defined as the patient’s experience of care coherence and enduring relationships to healthcare providers over time [6]. In Denmark, the state and the Ministry of Health have the overall responsibility for the healthcare system. The daily administration of the healthcare system is outsourced to regions and municipalities in Denmark. The five regions are responsible for the provision of hospital care, including emergency care and the services provided by the general practitioners (GP). The 98 municipalities are responsible for the local healthcare services such as home care, home nursing and rehabilitation outside the hospital, which an administration manages within a single municipality [7]. If a patient is referred to emergency care, as elsewhere in the healthcare system, coherent processes must be implemented [8]. The region’s vision is to create continuity of care in collaboration with the municipalities, especially when patients receive home or nursing care after hospitalization [9]. If a healthcare process includes acute hospitalization obstacles such as lack of communication, relatives’ involvement and not being involved in the process of one’s own care can hinder the continuity of care [10].

### Theoretical framework

Jürgen Habermas [1929-] is a German philosopher and sociologist and a representative of critical theory [11]. “Theory of communicative action” is one of Habermas’ most well-known theories regarding the theoretical perspectives of lifeworld and system. Through decades, Habermas’ critical theory has helped researchers gain a deeper understanding of modern healthcare practice and how health professionals maintain their professional integrity while navigating the system’s administrative requirements [12]. His theory can help explain why continuity of care is (or is not) experienced by patients.

Using Habermas terminology, healthcare belongs to the ‘system’. Hospitals, GPs and primary healthcare providers are different parts of this ‘system’. Symbolically generalized media such as money (e.g. appropriations) and power (e.g. administrative decisions and legislation) are used as a means to control the ‘system’. Patients’ social life belongs, according to Habermas, in the ‘lifeworld’ [13]. The patients’ lifeworld is characterized by communicative action, meaning that interaction in the lifeworld is based on a ‘consensual coordination of action’ [14]. Actors in the lifeworld of patients can achieve a common understanding through “communicative action” [13]. A lifeworld represents the social world, where patients organize their life within a private sphere. When the ‘system’ intrudes inappropriately or unpleasantly into the patients’ lifeworld, it disturbs the communicative action and the opportunity to achieve ‘consensual coordination of action’ is lost. This can contribute to loss of meaning, loss of sense of belonging and loss of identity [13, 14].

In our daily activities, we shift back and forth between our lifeworld and the system. A simple example of the lifeworld and the system interacting as a normal part of life is when patients visit their general practitioner to seek advice about a medical problem. Problems in communicative action occur when the reproduction in the lifeworld is not structured according to mutual understanding (‘consensual coordination of action’) but instead is structured according to money (e.g. appropriations), administrative decisions and legislation. Despite the best intentions in achieving a mutual understanding by communicating face to face, the system cannot ensure mutual understanding among all citizens at the same time. Instead, the system communicates decisions via rules, legislation and money, such as payment of fines as decided by elected representatives of people in parliament [13].

When communicative action and mutual understanding fails between a patient and professional, it may be because professionals base their decisions and conduct on the system’s rules rather than on a mutual understanding of the patient. Habermas anticipated that in these situations, the patient might experiences loss of meaning or loss of identity [13], leading to a lack of continuity of care in a healthcare process. When professionals extend their help beyond assigned tasks and rules, listening to a patient’s request and arguments and helping by following the patient’s wishes and needs, the professionals are engaged in communicative action with the patient.

Using the theoretical framework of Jürgen Habermas “Theory of communicative action”, this paper aims to explore why some acutely hospitalized multimorbid older patients do not experience continuity of care during their path in the Danish healthcare system [13].

## Methods

### Interviews

Patients admitted acutely to the emergency department at Hospital Sønderjylland were invited to participate if they were 65 years of age or older, had two or more chronic diseases, received home care service regularly and were legally competent. The first author selected the patients from the logistic system. The nurse responsible for the patient had to confirm that the patient matched the inclusion criteria and could participate. The gatekeeper (second author) informed and invited patients to the study, and if the patients agreed to participate, the interviewee (first author), informed them verbally and in writing about the details of the study. The interviewee did not know the nurses, but the gatekeeper is Chief Physician at the emergency department at the hospital, knew the nurses who participated. The patients that agreed to participate signed a consent form. The first interview took place at the hospital, and the second interview in the patient’s own home. The first author completed all interviews between January 2018 and August 2019 using two separate semi-structured interview guides (Supplementary file 1). Interviews were transcribed from audio records, and the program Nvivo (Nvivo quality data analysis software: QSR International Pty Ltd. Version 11) managed and sorted the data. Recruitment to the study ceased when a high and sufficient level of information power was attained. Information power is a tool developed to consider the sample size in qualitative studies. Information power uses the aim, the specificity of the patients, the use of theory, the quality of the interviews and the analysis strategy from the study to ensure an adequate number of patients. If these five items are broad more patients are needed, and if these items are narrow fewer patients are required. Our aim is broad, the characteristics of our patients are specific, we use theory to our findings, the quality of the interviews developed throughout the study, and we use the well-known content analysis to our findings [15].

### Evaluation of continuity of care

**Fig. 1:**
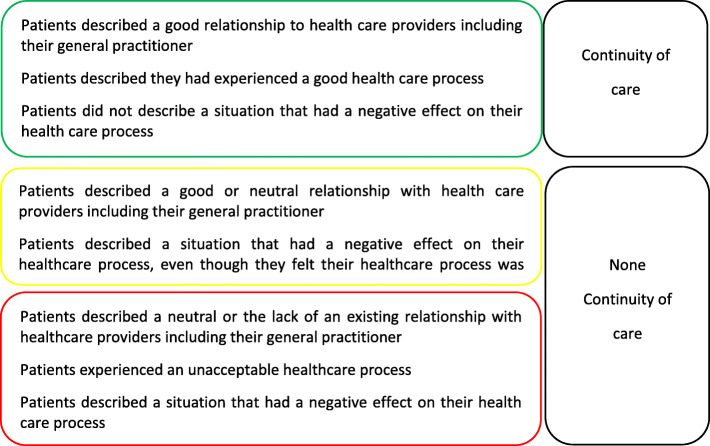
Evaluation of continuity of care in patients’ healthcare process

Prior to the formal analysis of the interviews, the authors evaluated if continuity of care was present in the patients’ healthcare process based on patient expressions. The healthcare process was coded green if continuity of care was evaluated as part of the process or coded yellow or red if continuity of care was evaluated not to be present in the process. A process was colour-coded yellow if the patient’s account indicated a lack of continuity of care or if a situation was experienced as negative, even though the overall process was reported as satisfactory. Healthcare processes were coded red if the patient’s own experiences of the healthcare process or continuity of care were inadequate. Figure 1 presents the evaluation strategy.

### Analysis

The analysis process used elements from Elo and Kyngäs approach to qualitative content analysis [16]. Unpublished observations by the authors use the same interview material, this data indicated that patients’ experienced obstacles to continuity of care (unpublished observations, Boye, LK., Mogensen, CB., Tanggard, PT., Waldorff, FB., Mikkelsen, TH.). By reusing the material from Elo and Kyngäs version of content analysis, it is possible to organize the data into a structured matrix. The use of a matrix in content analysis is specific for Elo and Kyngäs, and therefore we use their version of content analysis. This structured matrix enabled a specific coding to situations where the patients’ lifeworld and the system lacked ‘consensual coordination of action’ based on Habermas theory. In other words, we coded the situations where there were obstacles in a situation, e.g. Lack of communication, arguments or misunderstandings. We developed the matrix during the organizing phase, based on the knowledge we gained in the preparation phase of the content analysis, which means reading the interviews and applied theory, and coding the above-mentioned situations to gather data which was then was grouped into categories [16]. After the categorization, the material was analyzed based on Habermas theory of communicative action.

## Results

Of the twenty-two patients invited to participate in the study, fifteen completed both interviews. Seven patients were lost to follow up and did not complete the second interview. These patients were lost to follow up because they were not contactable, were readmitted or were too ill to participate, or had died. When the fifteen patients had completed the second interview, the authors agreed that adequate information power had been obtained. Demographic data and evaluation of continuity of care of the 15 patients are presented in Table 1. Seven patients evaluated to have experienced continuity of care, and eight patients were evaluated as not having experienced adequate continuity of care. Four of these eight patients had a relative that participated in the interview.

**Table 1:**
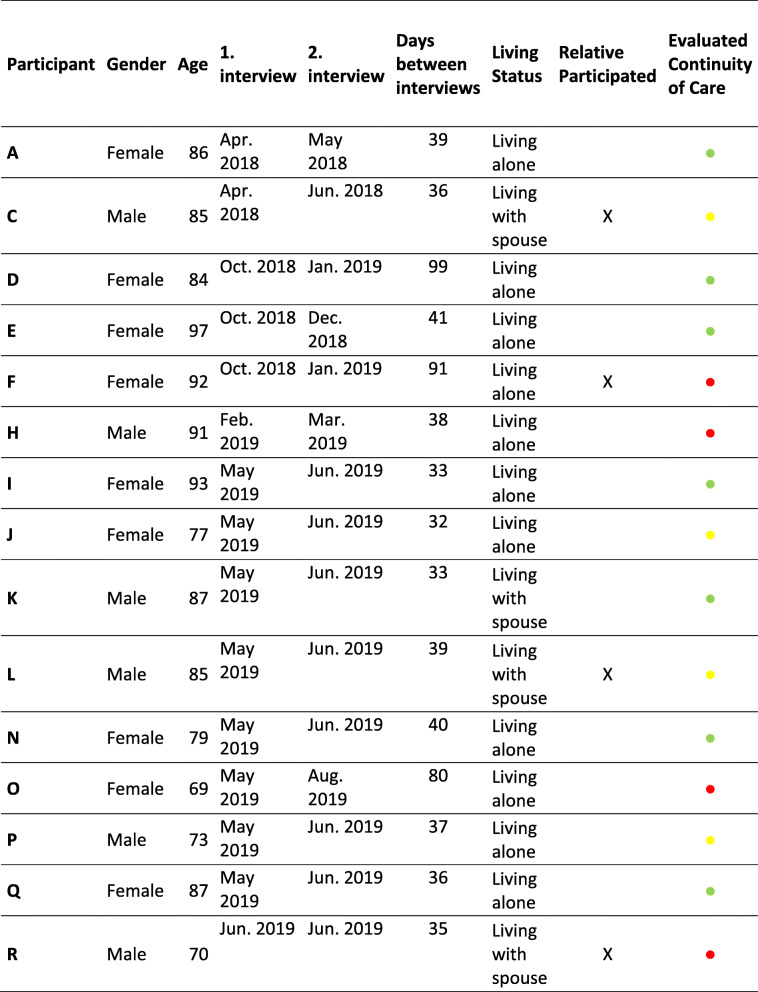
Patient information and the evaluation of continuity of care

After evaluation of the continuity of care, the interviews were analyzed using the structured matrix. Situations describing lack of ‘consensual coordination of action’, e.g. disagreements, unacceptable communication or lack of awareness in the situation, were coded into the structured matrix and grouped into the three categories: ‘Communication, frustration, expectation in the healthcare process’, ‘Relations to healthcare providers’ and ‘Objectification of the patients’. Table 2 elaborates on the condensation of the codes in each category.

**Table 2:** Structured matrix

	Communication, Frustration, Expectation in the healthcare process	Relations to healthcare providers	Objectification of the patient
Lack of ‘consensual coordination of action’	Understanding of what is possible in the care process and being frustrated Expectations were not aligned Changes in care were not communicated	No existing relationship to providers Busy staff	Feelings of not being involved in care Feeling like an object not a person Feelings of being a stranger in their own home

### Communication, frustration, expectations in the healthcare process

Continuity of care was evaluated as present in almost half of the study population’s course of treatment in the healthcare system. However, difficulty in achieving continuity of care was often experienced when patients did not understand the reasoning behind decisions and treatment plans. One patient explained:

“It is that a doctor comes in and asks you about some things, then ten minutes later another doctor comes, and he asks, God help me, about the same thing again. Then I think you could do that better (...) now the last time I was not involved in the round at all.” (O)

The feeling of not being involved in your own care is frustrating; the process does not ensure continuity of care and is an example of a lack of communicative action. Continuity of care is also challenged when the patient is referred to another home care group (e.g. a rehabilitation group instead of the usual home care group). After a relative had been in contact with the home care services administration, they explained:

“They did not know what to apologize for, because first it was the rehabilitation group, and I don’t know them at all. Someone came to start a rehabilitation process, and I almost lost it and said 'I think you should go' because this won’t work. Then she left. She could also hear that it was awful. I said 'she does not need to be rehabilitated - she just needs her regular care'” (F’s relative)

This example demonstrates the feelings of frustration, as this was not what they expected. Relatives are a resource for the patients in the healthcare system. When a relative feels that the patient is being neglected and information is not made available, it has an impact on how the patient views the system. Changes in the patient’s everyday life must be communicated to the patient to involve them in their own healthcare process and offer the opportunity for their own opinion to be expressed. Another example of lack of continuity of care is the relative who had been in contact with the home care services administration. The family member was discharged on a Friday and did not receive home care until Monday:

“We didn’t know what happened, because the hospital had arranged for someone to come, she already had home care, and when you are 92 and a lady, having two men come, whom she has never seen, she was terrified. They were from something called the rehabilitation group, so she should only have had her regular care” (F’s relative)

“Yes, I was like outside myself when they came, because I was sitting and showering myself. Then they took me in there onto the middle of the floor and started to dress me” (F)

These professionals solve the task they have been assigned according to the rules of rehabilitation. However, they should not have been assigned this function, and they failed to engage in communicative action, missing the opportunity to coordinate their actions with the patient and at the opportunity to discover they were performing the wrong task with this patient.

Communication about changes to everyday life is also relevant in the case of the next patient. He had received a hospital bed after being discharged, so the home care staff could perform their tasks safely. The first problem occurred with the arrival of the hospital bed

“They came with this bed the next day. I didn’t know I was getting one. I told them where to put the bed, but would they carry my old bed out? I couldn’t do (swearword) it myself. ‘No, we mustn’t. It doesn’t fit (swearword) well. But they did it anyway” (P)

This example demonstrates how the system engages in strategic actions to ensure a hospital bed is ordered for the patient but fails to inform the patient that it is arriving, why it was granted and why they cannot remove the old bed. However, these professionals engaged in communicative action, and they removed the old bed, despite their contradictory instructions.

A few days later, this patient was recovering well and could be washed in his own bathroom.

“Yes, last Thursday, I was up and ready to be washed. I suggested that we could do it in the bathroom and we did this for two days in a row. However, she (homecare provider) came back and said ‘I need to tell you something’. ‘If you’re able to be washed in the bathroom, the bed will be removed again’. What – you (swearing) can’t do that, I have sold mine, the other (bed). ( … ) I was (swearing) really upset.” (P)

The patient had not been informed that the hospital bed was a temporary solution whilst he was recovering. The hospital care bed had a significant influence on his quality of life, and the thought of losing it had consequences for his wellbeing. Home care services are not obligated to permanently grant the hospital bed to a patient despite it improving their quality of life. The patient must be made aware of the opportunities and limitations within a healthcare system. Although healthcare professionals are obligated to perform the tasks based on rules, they must engage in communication with patients to ensure they agree and understand the purpose and the scope of these tasks. Another study participant illustrated this in his journey from the hospital back to his own home.

“I told the taxi driver that I needed help to into my house. I explained to them that this task had been specifically requested by the hospital before the journey. The trainee driver said that they had not received any special request. I suggested they ring the hospital again. Whilst looking at his phone the trainee found the initial request. I repeated that we still had a problem because I needed to be lifted and supported into my house. The taxi drivers replied that they were not allowed to undertake that type of activity. I held my ground and repeated to them that they had had a specific request from the hospital to help me and they needed to find a solution - this was not my problem … ..When they tried helping, one of them couldn’t help at all because of back problems and the other couldn’t move much because of his stomach. The problem is that although I find this is completely unacceptable I can’t see where on earth I should send in a complaint”. (L)

The patient’s expectations of the journey home have not been met. The experience is particularly negative, as the patient had indicated his need for assistance whilst at the hospital. This is a good example of how communicative action is vital for the coordination of tasks, and without this, misunderstandings occur more readily. Coordination based solely on following guidelines and a ruleset leads to inadequate solutions and frustrated patients.

Requiring mobility and daily living aids from the municipality can also be a challenge for patients and relatives in relation to successful communication. This example demonstrates the challenges that occur when there are differences in expectations between the patient and the provider.

“I assumed that it (assistive devices) would arrive sometime last week. She rang the Friday before last and so I assumed it would come on one of the days last week. But what do I know! We don’t sit around at home waiting for them to come. We specifically told them that they had to ring before they came to make sure we were home. Things get messed up occasionally. Suddenly he turned up on Friday with the aid. I had told them that he had to ring before we came. We are not just sitting around waiting for him” (R’s wife)

This quote illustrates that the system coordinates among professionals whilst seemingly forgetting or neglecting coordination with the patients. Exclusion, whilst unintentional, can leave patients feeling more like a commodity needing to fit in with a professional’s schedule. Ultimately, this type of action leaves patients passive and inactive with reduced opportunities to how and when they want to spend their day. The family described above have a clear understanding of how the system works. When the system does not live up to these expectations, continuity of care is absent. Although we cannot draw a conclusion about the narrative above it is clear that the system reduces patients’ opportunities for autonomy.

### Relations to healthcare providers

Professionals in the health system are working under conditions that are regulated by law; This can pose an ethical dilemma as they cannot always take care of the patients in the most optimal way. Health professionals are paid to complete a single task, decided a priori by others based on rules and initiatives. Allowing these professionals into your lifeworld from the system can be challenging, as the predefined job often does not require mutual understanding through communicative action. Besides the predefined task or job may be completed by a variety of different types of health care professionals.

“I do not talk much with them, they do what they have to do, it goes fast. It depends on who comes. We take it as it is, I accept it ( … ) I want her (name of home care provider), she’s the one who comes most and she’s also quick to get it all done, they have to move on they are so busy” (J)

Most patients feel most comfortable if they are familiar with the health professionals who come to their home to perform specific tasks, it makes them feel less like they are welcoming strangers to their home. A patient’s statement “I accept it”, indicates, that help is required, but acceptance is not the same as feeling highly satisfied.

“She would (swear word) call the head nurse and everything and stuff like that. She would not take responsibility so I told her that I would take the responsibility ( … ) I think that it is good, they have some rules to follow. But I think when we say such and such, it is not for fun, then I think they to respect it, but yes, I also listen to what they say, but afterwards I do it anyway, because it cannot be right”(P)

This quote demonstrates well the differences between the system and a patient’s lifeworld. While the healthcare worker, on the one hand, refers to her/his superiors in the system, the patient, on the other hand, expects the healthcare worker to engage in communicative action. When realizing that the patient’s wishes cannot be fulfilled through communicative action, the healthcare worker does not have the authority to decide upon a different level of service.

### Objectification of the patient

Unwell patients needing help from others creates a situation of vulnerability, and for some patients, they feel more like an object than a person. This feeling also relates to the other categories where most patients felt misunderstood, frustrated or not involved in their own care. When patients need help, they need to adjust to the ‘system’, which can be difficult, especially if you feel unimportant. One patient expressed it clearly:

‘One feels somewhere that one is insignificant in that system’ (R)

A patient and his wife had the feeling of sitting at home and waiting for people to come and help them as if they were not significant. A similar feeling is reported from other patients when they describe being a stranger in their own home or feeling like an object placed in a room in a hospital. It can also be after an appointment with a GP that prescribes more medicine rather than listening and understanding the problem. These situations do not contribute to the continuity of care. A patient experiences lack of continuity of care, when healthcare providers fail to engage in communicative action, and the patients suffer from loss of meaning or loss of identity.

Contrarily, a patient who experienced continuity of care expressed:

‘Yes and so all the time you have the feeling that they are interested in us’ (K)

When the ‘system’ provides continuity of care, patients feel involved, listen to and guided through the system, despite meeting with many healthcare professionals increasing the risk of breaking the continuity of care. If patients feel ‘they are of interest’, it is most likely that continuity of care is present.

## Discussions

Our findings indicate that almost half of the patients interviewed experience consensual coordination of action resulting in a positive experience with the healthcare system. Nevertheless, the other half of the study population experienced a lack of continuity of care, which appeared to occur primarily with short specific healthcare activities that were granted by one type of health care professional but conducted another, e.g. the taxi driver or the men delivering a hospital bed. These professionals have a straightforward task or service to perform, which does not require or necessitate prior background knowledge. Nevertheless, these professionals and their interaction with patients are also essential in creating continuity of care. It is vital that these types of professionals also engage in communicative action with patients to ensure coordination of care and guard a patients’ wellbeing. However, the results of this study give rise to optimism, as many healthcare professionals already engage in coordinating action with the patients, e.g. mutual understanding of treatment or discharge concerning their needs is often achieved with the patients. According to Habermas’ lifeworld and system framework, the content analysis contributed to an increased understanding as to why some acutely hospitalized older people do not experience continuity of care. Patients cannot experience continuity of care if there is no consensual coordination of action between the patient and the professional. Consensual coordination of action will not occur if the patient does not understand the availability of options in their care process, if they feel frustrated, if their expectations are not met or if changes in care are not communicated. Strained relationships with healthcare providers or if patients feel the healthcare system is too busy also contributes to a lack of continuity of care. According to Habermas, patients not involved in their own care, feels like a stranger in their own home or feel unimportant reflect a colonization of the lifeworld, which affects the patients’ sense of belonging and identity [13]. The colonization of the patients’ lifeworld can help explain why some patients experience obstacles that affect continuity of care.

Such deficits in communication may affect the patients trust in healthcare services. There is considerable evidence that patients involved in their own care with a high level of trust for their healthcare providers [10] will have a direct, positive influence on health outcomes and patient satisfaction [17]. The legitimacy to provide care for people is based on authorization and legislation from the Danish Health Act [18]. This legitimacy must not be used to exercise power [19], and healthcare services must ensure consensus and communicative action. The Healthcare Act requires that healthcare providers ensure respect and integrity for individual human beings and opportunities for self-determination through a transparent healthcare system (Chap.1, §2.) [18]. This research demonstrates how just a few unfortunate events in the health care process often connected with transfer between sectors feeds a lack of trust in the system. In some cases, professionals not authorized under The Health Act undermine this trust, e.g. taxi drivers/mobility aids delivering driver. These mistakes may also occur because of the different types of incentives found between sectors and healthcare providers [20].

Patients assume healthcare providers act in their best interest [10, 21], but this notion is challenged when patients feel frustrated or unable to understand how the system works or why changes are made. Continuity of care is vital but challenging to maintain in a healthcare process involving many transitions [6, 22, 23]. Continuity of care could be improved if the non-healthcare frontline workers with relative minor tasks were instructed to engage with patients to understand the background behind their task, ensuring that the patient agrees with the need, the tasks and the outcomes.

### Strength and limitations

Our study has three main strengths that increase the reliability of the findings. Firstly, all patients were interviewed twice, providing an opportunity to elaborate on findings and comments from the first interview. Secondly, the preparation phase to analyze the data was conducted in a former study and thereby, the content analysis was conducted with a strong foundation. Finally, the study was designed with 15 patients interviewed twice, ensuring validity based on the number of participants, quality of the interviews and recruitment of participants resulting in high information power [16]. An additional strength of this study was applying Habermas’ theoretical perspective to the matrix, which allowed us to make a critical analysis. The first limitation was that there was a single isolated evaluation of continuity of care rather than a measurement of this phenomenon [6]. However, we conducted this evaluation in collaboration, and there was consensus regarding the result of the evaluation. The second limitation was that information between the days from discharge to the second interview may have influenced the findings. Finally, exploring how healthcare providers viewed continuity of care would have been an interesting perspective to include in this study.

### Clinical implications

The clinical implications include a need for the healthcare sector to focus on non-healthcare professionals who play a role in providing care for patients’ needs. Healthcare professionals must communicate and listen to patients ensuring that patients and relatives understand their options. In addition, our findings imply that the healthcare sectors need to be aware of each other’s services to reach a common understanding of each other’s expectations.

## Conclusions

For acutely hospitalized older multimorbid patients, obstacles to the continuity of care mainly occurred in the transition between healthcare sectors, particularly in situations where non-healthcare professionals perform a task. These obstacles often occur when mutual understanding by communicative action with the patient is neglected or forgotten. In conclusion, healthcare providers and all kinds of professionals in contact with patients must engage in communicative action with the patients to secure continuity of care. These results justify a focus on improving the mutual understanding between the patients and the professionals in the transition between healthcare sectors since this will most likely benefit continuity of care for the acutely hospitalized older multimorbid patients.

## Supplementary Information

**Additional file 1.**

## Data Availability

The datasets generated and analyzed during the current study are not publicly available due to regulations from The Danish Data Protection Agency but are available from the corresponding author on reasonable request.

## Abbreviations

GP: General practitioner
