# Supplementary material for: ‘One feels somewhere that one is insignificant in that system’ – older multimorbid patients’ between lifeworld and system in healthcare
Source: BMC Geriatr. 2021 Jun 29;21:397. doi: 10.1186/s12877-021-02348-x (PMC8243446; doi:10.1186/s12877-021-02348-x)
Supplement: Supplementary file 1 — Additional file 1. [file 12877_2021_2348_MOESM1_ESM.pdf]

Supplementary files:

'One feels somewhere that one is insignificant in that system' – Older multimorbid patients' between lifeworld and system in Healthcare

Lilian Keene Boye (1, 2), Christian Backer Mogensen (1, 2), Pernille Tanggaard Andersen (3), Frans Boch Waldorff (4, 5), Thorbjørn Hougaard Mikkelsen (1, 2, 4)

Affiliation:

1. Emergency department, Hospital Sønderjylland, Aabenraa, Denmark.
2. Research unit of emergency medicine, Department of Regional Health Research, University of Southern Denmark, Odense, Denmark
3. Unit of Health Promotion, Department of Public Health, University of Southern Denmark, Esbjerg, Denmark.
4. Research unit of General Practice, Department of Public Health, University of Southern Denmark, Odense, Denmark.
5. Section of General Practice, Department of Public Health, University of Copenhagen, Copenhagen, Denmark

Corresponding author:

Lilian Keene Boye

Hospital Sønderjylland,

Kresten Philipsens vej 15, indgang F

6200 Aabenraa, Denmark.

Lilian.keene.boye@rsyd.dk

Supplementary file 1: Interview guides.

Semi-structured Interview guide, 1th interview.

Introductory remarks:

Thank you for your interest in participating in the project. I would like to tell you a little about myself and my project. Repeat the introduction to the project, number of interviews, what they contribute to in relation to the project. Information of audio recording.

Review of consent with the patient, signing of consent form.

(Start audio)

The purpose of today's interview

- Getting to know and hear more about your story
- An informal conversation - I ask questions and you answer, there is no right or wrong.
- I may well repeat your answers to make sure I understand you correctly.

Questions to support the interview:

I would like it if you could tell me more about your health care process? What chronic diseases do you have? How often have you been in contact with healthcare professionals?

Why were you were admitted to hospital? Who decided you needed to be admitted, who helped you, what were you responsible for yourself? What has happened whilst you have been hospitalized?

Can you explain briefly about how much help you get at home - And what sort of help? Do you think you get enough help?

Who do you see as your primary caregivers - both private as well as professionals?

Are you already thinking about when you get discharged? Will you need more help?

Thank you, for your time and participation, I will contact you when it is time for the next interview.

Semi-structured Interview guide - 2nd interview

Introductory remarks:

Thank you for allowing me to interview you again. The last time we talked, you were in hospital. I would like to summarize why I want to talk to you again today. Repeat purpose of the project.

(Start Audio)

The purpose of today:

We are going to talk a little again about the same things as last time, but I will also ask about how you feel now, after being in hospital.

It's still an informal conversation – so there are no right or wrong answers.

I also will follow up on some of the things you said last time.

I want to hear about you, your story and your opinions. I would like you to respond from your own point of view, not just from what you think I would like to hear.

Questions to support the interview:

Can you tell me what's happened since the last time we talked?

How did you experience being in hospital?

Is it a relief/good/comfortable being home now?

Have you needed extra help?

How are you feeling generally? Better?

How was your experience of the hospital, the municipality and your doctor working together?

How did they collaborate?

Has your healthcare process made sense to you?

Do you feel that there were continuity of care in your healthcare process?

When I ask about your continuity of care and collaboration between hospital, municipality and your own doctor, what do you understand with the expression ‘continuity of care’?

Is there anything you have had questions or were concerned about?

In the healthcare process, what has been your role?

Have you been involved in decisions that needed to be made?

How much responsibility do you want in relation to decisions and involvement?

Have you received help from your relatives?

Have they had any responsibility?

Have they been involved in decisions regarding your situation?

If you were to avoid going to hospital again, what should be done? How could it be avoided?

I've talked a lot about creating better continuity of care in the healthcare system for older people, how can we best involve you in this process?

Additions after pilot-testing.

What is the most important thing for you in a health care process such as the one you have just been through?

Are there any specific small things that have had a positive or negative influence?

Have you felt listened to?

Thank you, for your time and participation in the project. If you have any question, do not hesitate to contact me.
